# Is overpromotion of emergency contraceptives misdirecting youth away from condom culture?

**DOI:** 10.4103/2589-0557.69005

**Published:** 2010

**Authors:** Karun Jain, Jami Swathi

**Affiliations:** Department of Orthopaedics, JSS Medical College and Hospital, Mysore - 570 004, India; 1 Department of Medicine, Stanley Medical College, Chennai-600 001, India

Sir,

News papers, magazines or electronic media, now-a-days we see almost everywhere the advertisements of emergency contraceptives. Among the general public, these emergency contraceptive pills are promoted as the remedy to the tension of pregnancy if sexual exposure was unprotected. Although the intention of the drug companies and government agencies may be right and justified, but unrestricted access to emergency contraception in advance may act as a psychological pull for youngsters to have sexual contact without employing the usual contraceptive measures.

We performed a simple questionnaire-based analysis of 250 sexually active males, aged 21-35 years, for 6 months, from September 2008 to March 2009, in Mangalore city. The main reason to have only the male group in our study is that our society is mainly male dominant and that they still have the upper hand during intercourse over their female better-half. We found that the most common mode of contraceptive measure was condom followed by coitus interruptus. During the last 6 months of their sexual life, they had used emergency contraceptive pills on an average of eight times per male. But, in only 1.2 times per male, these pills were actually used after a forgotten unprotected intercourse and during the remainder of the times, they had convinced their partner to use emergency contraceptive pills later and intentionally had sex without any protection. The most common reason advocated for unprotected intercourse was to have complete and uninterventional sexual pleasure. Surprisingly, we found that not every time our study group males had to convince their partner for emergency contraceptive pills later. On an average of three times per male during 6 months, their female partner volunteered to have unprotected intercourse. In our study, 76 males had multiple sex partners and 29 males had visited professional sex workers. One hundred and forty-three males were concerned of only their sexual pleasure and were not much aware of possible sexually transmitted diseases that can occur during unprotected intercourse, especially among the high-risk males like multiple sex partners and those visiting professional sex workers.

Among the 208 males who have made their partner use the emergency contraceptive pills, it was found that they were keen to use this contraceptive measure routinely irrespective of knowing the fact that it is an emergency pill and not a regular one. Weaver *et al*.[1] also concluded that with an unrestricted access of these pills, some women may substitute emergency contraception for their usual contraceptive methods. We have not measured the contraceptive failure rate as it was not the objective of our study.

Increased access to emergency contraceptive pills has surely enhanced its use, but the existing literature is unable to justify and also creates confusion regarding the usefulness of these emergency contraceptive pills in reducing the unintended pregnancy rates.[2–4] We still do not have evidence-based research of a possible increased rate of sexually transmitted diseases like human immunodeficiency virus, hepatitis B and many more diseases with the overuse of emergency contraceptives.[5] Further research is needed to explain this finding and to define the best ways to use emergency contraception to produce a public health benefit.
